# SUBJECTIVE SOCIAL STATUS AND DEPRESSION AMONG KOREAN OLDER ADULTS: FORMAL SOCIAL ACTIVITIES AS MEDIATOR

**DOI:** 10.1093/geroni/igad104.3606

**Published:** 2023-12-21

**Authors:** Junmin Park, Yeonjung Lee

**Affiliations:** Chung-Ang University, Seoul, Republic of Korea; Chung-Ang University, Seoul, Republic of Korea

## Abstract

Previous studies have examined an association between depression and socioeconomic factors such as education, economic status, and social support among older adults. It is widely acknowledged that socioeconomic status (SES) plays a pivotal role in the manifestation of depression. However, subjective social status (SSS) representing the subjective perception of the accumulation of life experiences and the associated overall deprivation may hold even greater importance in older adults. Especially, statistics in Korea shows that most people are likely to consider themselves in middle class regardless of their objective financial status. Therefore, the objective of this study is to examine whether subjective social status is associated with depression in later life as well as how participation in formal social activities (i.e., religion/social gathering, leisure/cultural/sport activity, reunion/hometown alumni, volunteering, party/civil society organization/advocacy group) mediate this association. Multiple regression analyses and Baron & Kenny (1986) analytical approach were applied using a panel data from the 2020 Korean Longitudinal Study of Ageing. Selected sample include those who are aged 65 and older (n= 4,405). Results show that there is a negative association between SSS and depression. SSS was positively associated with formal social activities. Additionally, formal social activities have significant impact on mitigating depression. Participation in formal social activities partially mediates the relationship between the SSS and depression. Findings have implications for promoting an opportunity for older adults to participate in some formal types of social participation. Furthermore, additional research investigating the factors that can augment SSS should be warranted.

